# Two cases of carfilzomib‐induced thrombotic microangiopathy successfully treated with Eculizumab in multiple myeloma

**DOI:** 10.1186/s12882-020-02226-5

**Published:** 2021-01-18

**Authors:** Michael Rassner, Rebecca Baur, Ralph Wäsch, Mario Schiffer, Johanna Schneider, Andreas Mackensen, Monika Engelhardt

**Affiliations:** 1 Department of Medicine I (Hematology, Oncology and Stem Cell Transplantation), Medical Center, Faculty of Medicine, University of Freiburg, Hugstetter Strasse 53, D-79106 Freiburg, Germany; 2 Department of Internal Medicine 5 - Hematology/Oncology, University Hospital of Erlangen, Erlangen, Germany; 3 Department of Internal Medicine 4 - Nephrology, University Hospital of Erlangen, Erlangen, Germany; 4 Department of Medicine IV, Medical Center, Faculty of Medicine, University of Freiburg, Freiburg, Germany

**Keywords:** Multiple myeloma (MM), Thombotic microangiopathy (TMA), Eculizumab, Carfilzomib, Case report

## Abstract

**Background:**

Treatment with proteasome inhibitors like carfilzomib in patients with multiple myeloma (MM) can induce thrombotic microangiopathy (TMA) characterized by neurological symptoms, acute kidney injury, hemolysis and thrombocytopenia. Successful treatment with the monoclonal antibody eculizumab was described for these patients, but reports of ideal management and definitive treatment protocols are lacking.

**Case Presentation:**

The first case describes a 43-years-old IgG-kappa-MM patient that developed TMA during the first course of carfilzomib-lenalidomide-dexamethasone (KRd) consolidation after autologous stem cell transplantation (ASCT). In the second case, a 59-years-old IgG-kappa-MM patient showed late-onset TMA during the fourth and last cycle of elotuzumab-KRd consolidation within the DSMM XVII study of the German study group MM (DSMM; clinicalTrials.gov Identifier: NCT03948035). Concurrently, he suffered from influenza A/B infection. Both patients had a high TMA-index for a poor prognosis of TMA. Therapeutically, in both patients plasma exchange (TPE) was initiated as soon as TMA was diagnosed. In patient #1, dialysis became necessary. For both patients, only when the complement inhibitor eculizumab was administered, kidney function and blood values impressively improved.

**Conclusion:**

In this small case series, two patients with MM developed TMA due to carfilzomib treatment (CFZ-TMA), the second patient as a late-onset form. Even though TMA could have been elicited by influenza in the second patient and occurred after ASCT in both patients, with cases of TMA post-transplantation in MM being described, a relation of TMA and carfilzomib treatment was most likely. In both patients, treatment with eculizumab over two months efficiently treated TMA without recurrence and with both patients remaining responsive months after TMA onset. Taken together, we describe two cases of TMA in MM patients on carfilzomib-combination treatment, showing similar courses of this severe adverse reaction, with good responses to two months of eculizumab treatment.

## Background

Thrombotic microangiopathy (TMA) is a rare cause of anemia in multiple myeloma (MM) patients, but can occasionally be elicited by common anti-myeloma drugs, especially by proteasome-inhibitors (PI) like carfilzomib (CFZ) [1–3]. Reports about TMA in MM induced by CFZ and optimal therapeutic approaches are scarce. Recently, single case reports described successful treatment of TMA in MM with the terminal complement pathway inhibitor eculizumab [1, 2, 4–6]. Definitive treatment recommendations, however, are lacking. Below we describe two cases of CFZ-induced TMA, that were successfully treated by eculizumab and provide evidence about precise therapeutic work-flow and possible interventions.

## Case 1

**Fig. 1:**
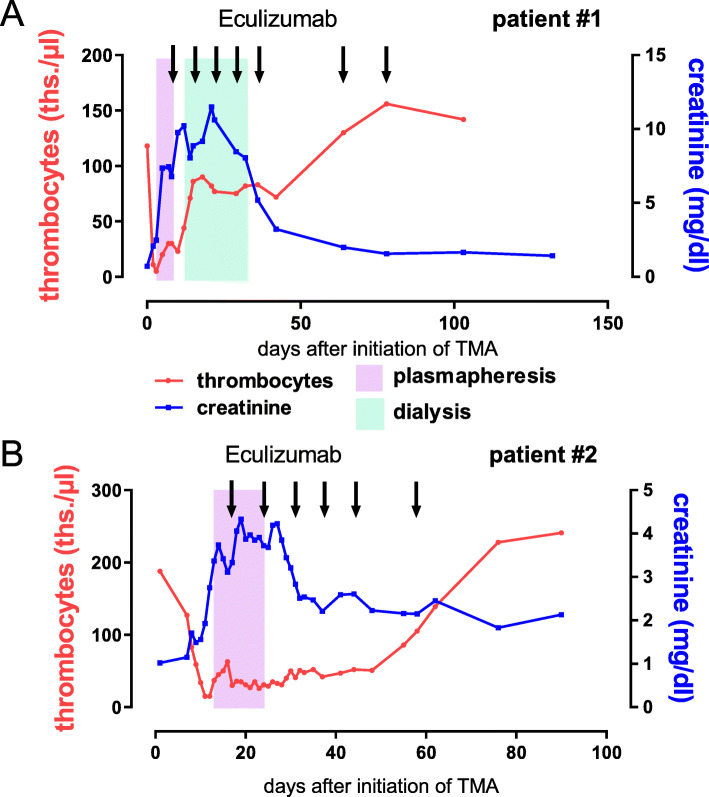
Course of CFZ-TMA in the two patients. **a** Case #1: This patient developed acute kidney injury, fever and pulmonary hemorrhage during the first consolidation with carfilzomib, lenalidomide and dexamethasone (KRd) after ASCT. Laboratory findings (thrombocytopenia) and schistocytosis of 11‰ were consistent with thrombotic microangiopathy (TMA). Therapeutic plasma exchange (TPE) was immediately initiated, but shortly after dialysis was necessary. As blood values did not improve, we finally administered eculizumab for a total of seven doses and saw a rapid response in LDH, platelet number and kidney function with our patient being still in PR. **b** Case #2: This patient, who is treated in the DSMM XVII study (Arm A: Elotuzumab-KRd) developed head ache, malignant hypertension and laboratory distortions (thrombocytopenia, increase of LDH and acute kidney injury) consistent with TMA. After a short period of TPE, eculizumab was infused (total of six infusions). Blood values improved but kidney function remains decreased

**Fig. 2:**
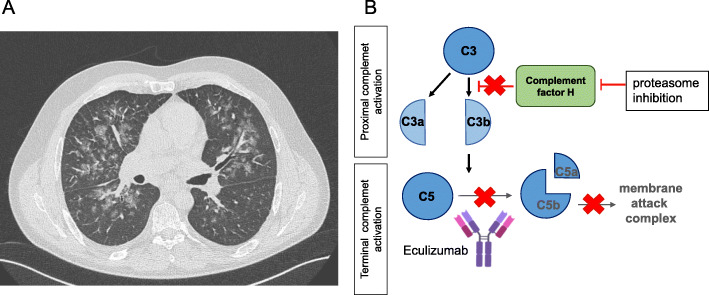
**a **Chest-CT in patient case 1, d1 of CFZ-TMA onset: Bipulmonary extensive ground-glass opacities in all lung fields, mostly omitting the periphery and predominantly occurring in the right lung. **b** Mechanism of CFZ-TMA: Within the proximal complement activation C3 is cleaved by the C3 convertase to anaphylactic 3a and C3b. C3b then cleaves C5 to C5a and C5b, the latter together with C6-C9 forming the membrane attack complex (MAC) within the terminal pathway. Activity of the C3 convertase is regulated by complement factor H and regulated proteins. Proteasome inhibition blocks CFH [11]. The monoclonal antibody eculizumab counteracts the resulting overactivation of C3b by binding C5

A 43-year old male patient was diagnosed with IgG-kappa-MM in August 2018. At admission, we found extensive osteolysis and an acute kidney failure with a serum-creatinine of 4.2 mg/dl [0.67–1.17] (GFR 15 ml/min; MDRD) without previous history of impaired kidney function. With highly elevated serum free kappa light chains (kappa-SFLC) 3645 mg/l [0.35–15.10] and 4.2 g/d [< 0.15] urinary protein excretion, in the absence of albuminuria, cast nephropathy was assumed as the most likely cause of acute kidney failure. Since there was no sign of further organ injury, amyloidosis seemed unlikely, as well MIDD (monoclonal immunoglobulin deposition disease), which is often accompanied by amyloidosis and presents with albuminuria. The type of renal injury was not further determined by kidney puncture. The M-protein was elevated with 37 g/l, IgG 52.3 g/l [6.1-16.16], k/l-ratio 864 [0.46-4], hemoglobin (Hb) 9.5 g/dl [13.5–17.5], β2-microglobulin 8.7 mg/dl [0-2.34], serum calcium and LDH were normal. The bone marrow plasma cell (BMPC) infiltration was 15%, FISH excluded high-risk cytogenetics. Following the ISS grading system, we diagnosed an IgG-kappa-MM stage III (Durie & Salmon stage IIIB) and symptomatic myeloma with 3/4 CRAB criteria (anemia, renal impairment, bone lesions). The revised myeloma comorbidity index (R-MCI: 1/9 = fit/low-risk) revealed a fit patient [7–9]. Due to the CRAB-criteria, specifically renal impairment, chemotherapy was initiated with one cycle of bortezomib, doxorubicin, dexamethasone (BAD) followed by two cycles of bortezomib, cyclophosphamide, dexamethasone (VCD) after improvement of the kidney function and resolved urinary proteinuria. Since M-protein reduction was insufficient (21.4 g/l), the regimen was switched a second time to CFZ, lenalidomide and dexamethasone (KRd). After two cycles KRd, a partial remission (PR; M-protein 5.3 g/l, IgG 6.4 g/l) was achieved and high-dose melphalan (200 mg/m²) chemotherapy with autologous stem cell transplantation (ASCT) was performed. On day (d) 42 post ASCT IgG levels were increasing again (21.1 g/l). Based on the distinct improvement of the myeloma markers after KRd and a missing additional response to ASCT, our MM-tumor board decided to apply three consolidation cycles of KRd. In May 2019, the 3rd KRd cycle (1st consolidation cycle after ASCT) was initiated. At d2 of CFZ, the patient developed fever, dyspnea and malaise. Platelets were 11Tsd/µl, Hb 10.8 g/dl, LDH 1836U/l, haptoglobin < 150 mg/l, unconjugated bilirubin 2.7 mg/dl, the creatinine rose from 0.72 to 2.07 mg/dl [0.67–1.17] (GFR > 60 to 35 ml/min) (Fig. 1a) and urinary proteinuria increased from normal values to 52 g/l [< 0.135]. In the peripheral blood smear, 11‰ schistocytes were detected and an altered complement profile with reduced C3 and C4 levels was found (C3 77.6md/dl, C4 9.9 mg/dl). TMA-Index (LDH/platelets ratio) was elevated to 166. These findings (schistocytes > 10‰, elevated creatinine and TMA-Index) have been described as poor prognostic factors for transplant associated-TMA (TA-TMA) [10]. Of note, two days earlier, lab results had been normal (Fig. 1a). Chest CT scan showed bilateral infiltrates, matching pulmonary hemorrhage (Fig. 2). Due to the acute development of hemolysis, together with acute kidney injury, unsuspicious ATAMTS13 activity (61%) [50–110], normal ADAMTS13 antigen (0.48 IU/ml) [0.35–1.2] and direct Coombs test being negative, we suspected medication-induced TMA. Therapeutic plasma exchange (TPE) was initiated by replacement with fresh frozen plasma (FFP) on a daily regimen and on account of declining kidney function with development of acute kidney injury stage 3 according to the acute Kidney Injury Network (AKIN), dialysis was necessary one day later. After six days of TPE neither LDH nor platelets improved. Assuming CFZ-induced TMA, we administered eculizumab (900 mg) along with meningococcal vaccination and initiation of antibiotic prophylaxis. Five days later, LDH declined to 818U/l and platelet counts improved to 58Tsd/µl without transfusions. We continued eculizumab (900 mg) weekly. After the fourth application, dialysis could be discontinued. Platelet counts normalized within two months after treatment initiation of eculizumab and there were no further signs of TMA, thus eculizumab was terminated (Fig. 1a). One year after TMA diagnosis and KRd consolidation, our patient remains in PR of his MM and CR of the CFZ-induced TMA.

## Case 2

The second 59-year-old male patient was diagnosed with IgG-kappa-MM in 11/2018. CT scan revealed osteolysis of the sacrum, pelvis and vertebrae. Calcium and creatinine were normal and the Hb was slightly decreased (13.3 g/dl). IgG was 38.4 g/l, k-SFLC 182 mg/l, k/l-ratio 60.5. BM biopsy revealed 40% PC infiltration. Cytogenetics were favorable (trisomy 5p15, 9q22, 11q13 und 15q22). The ISS and R-ISS were both I, Durie & Salmon stage was IIIA and symptomatic MM was diagnosed with 1/4 CRAB symptoms. The patient was classified as fit for ASCT (R-MCI-score: 2/9 = fit) [7–9]. We included the patient into the DSMM XVII study of the German study group MM (DSMM; clinicalTrials.gov Identifier: NCT03948035). Induction with elotuzumab, CFZ, lenalidomide and dexamethasone (E-KRd, 6 cycles) and ASCT with melphalan 200 mg/m^2^ were performed and due to repeated respiratory infections (boca- and corona (CoV NL 63) virus) dose reductions of elotuzumab and lenalidomide were executed. Before ASCT, kappa-SFLC decreased to 12.8 mg/l (vgPR).

In 01/20, within the last (4th) cycle of E-KRd consolidation, the patient developed fever and acute kidney failure with creatinine 1.7 mg/dl (GFR 40 ml/min, MRDRD; baseline-creatinine: 1 mg/dl). Prerenal genesis was assumed (fractional sodium excretion < 1%) and fluids were infused. However, creatinine elevation did not resolve finally reaching acute kidney injury stage 3 according to AKIN and proteinuria reached 6.2 g/d. The patient developed headache and high blood pressure (**BP** 220/105 mmHg). Platelets and Hb gradually decreased (Fig. 1b), haptoglobin was < 5 mg/dl. In the peripheral blood smear, 44‰ schistocytes were visible; ADAMTS13 activity and antigen were normal (58% and 0.40 IU/ml, respectively) and Coombs test negative. Blood culture additionally showed Staphylococcus epidermidis due to port infection and throat swab detected influenza A (H3N2) and B.

Since TMA was diagnosed (LDH/platelets ratio 88.3), high-dose glucocorticoids and TPE were applied (before completion of diagnostics). Without substantial improvement, five days after first plasmapheresis our patient received eculizumab (Fig. 1b), starting at 900 mg. A total of 11 sessions of plasma exchange at a daily basis were performed with FFP as replacement (Fig. 1b). Parameters of hemolysis rapidly improved, so that glucocorticoids could be expeditiously reduced and eculizumab was terminated after four cycles with 900 mg/weekly and two doses with 1200 mg/every two weeks (Fig. 1b).

In 05/19, the patient showed VGPR of his MM, the BMPC infiltration even CR (polyclonal PCs of 5%) and gradual renal function improvement (eGFR improved from 10 to 32 ml/min). Maintenance therapy with reduced doses of elotuzumab-lenalidomide (E-R: lenalidomide with 5 mg d1-28) was successfully initiated and TMA has remained in CR.

## Discussion and conclusion

Hemolysis is a rather rare cause for anemia in MM [12]. When anemia is accompanied with thrombocytopenia, acute kidney injury, high blood pressure, neurological or unexplained extrarenal symptoms TMA should be considered. TMA includes a group of etiologically diverse diseases: Primary TMA, which include atypical HUS (aHUS) or thrombotic thrombocytopenic purpura (TTP) and secondary TMA and infection-associated TMA, involving Shiga-toxin-producing Escherichia coli-associated hemolytic uremic syndrome (STEC-HUS) [6, 13, 14]. TTP, in contrast to other TMA forms, is defined by decreased activity of the vWF-cleaving enzyme ADAMTS13 [6]. Unlike “typical” HUS, which is triggered by infections with STEC, complement mediated TMA is driven by uncontrolled complement activation and affected patients frequently harbor mutations in complement regulatory proteins (Fig. 2b) [15]. Secondary TMA is elicited by medication, malignancy, pregnancy, malignant hypertension, transplantations, infections or associated to autoimmune diseases [6, 10, 12–14]. As reviewed recently [6], TMA in MM can be elicited by ASCT- or allogeneic-SCT, MM progression, or anti-myeloma therapy. Drug-induced TMA (DITMA) may occur in MM due to PIs bortezomib or CFZ [3, 16, 17]. CFZ-TMA can appear early after onset of the drug, but also as late as two years after first administration [1, 2]. CFZ-TMA is probably driven by complement activation of the alternative pathway in terms of complement-mediated TMA [2–4, 18]. Blasco et al. described three cases in which plasma samples from patients with CFZ-TMA, led to depositions of complex membrane attack complex (C5b-9) of on endothelial cells, suggesting a complement overactivation [4]. Supporting this assumption, proteasome inhibition has been found to block Factor H, consequently leading to complement activation (Fig. 2b) [11]. Bortezomib-induced cases of TMA are attributed to decreased activity of ADAMTS13 [19, 20]. However, the majority of bortezomib-induced cases of TMA/TTP had only slightly decreased ADAMTS13 activity and PI-induced TMA in MM might be generally based on pathological overactivation of the alternative complement pathway rather than TTP [6]. In patient #2, TMA was either induced by CFZ or influenza A or B [15, 21]. Also a combination of both preexistent vascular endothelial injury due to CFZ and transient complement activation during influenza infection was eagerly discussed within our group. In this respect, eculizumab could be effective in patients treated with concurrent PI treatment, infection and TMA.

In patient #2, in contrast to patient #1, C3 and C4 levels were normal; CH50 and C5b-9, the terminal pathway of the complement pathway were, however, not assessed. The fast response to eculizumab and the lacking effect of TPE seemed to argue for pathological complement activation, but it cannot be ruled out that the classical or the alternative complement pathway was involved. Importantly, both patients developed TMA several weeks after ASCT. Since TA-TMA was described to occur in a complement-dependent manner, like complement mediated TMA [10], a combination of TMA-triggers is likewise conceivable.

Taken together, several factors might have induced TMA in our patients: ASCT, genetic predispositions, viral infections and the underlying CFZ treatment. Thus, definite causality could ot be entirely proven. The major criticism of our study may be missing additional *in vitro* studies of complement activation and the unavailability of renal biopsies for further diagnostics. Kidney biopsies had been discussed interdisciplinary in our weekly MM tumor board, however, in the acute clinical setting, the potential biopsy risk, especially bleeding, was judged to outweigh potential benefits and on top, both patients had declined kidney biopsies.

In respect to therapy for TMA in MM, definitive clinical practical guidelines do not exist [6]. For DITMA, the causative drug must be discontinued promptly, high-dose glucocorticoids are given and plasmapheresis is performed [2, 6]. In CFZ-TMA, successful treatment with eculizumab, a terminal complement pathway inhibitor, was described in six cases to our knowledge [1, 2, 4, 5, 22]. Nevertheless, reports of TMA in MM remain scarce, and the definitive treatment regimen as well as definitive duration of treatment are unclear [6]: Portuguese and Lipe (2018), Moliz et al. (2019) and Blasco et al. (2020) administered eculizumab for up to three weekly doses [2, 4, 22], whereas Bhutani et al. (2020) for four months [1]. In our two cases, only after eculizumab initiation, hemolysis and creatinine levels improved impressively (Fig. 1A,B), plus the application period of two months of eculizumab was efficient without relapsing TMA, thus appears possible to be shorter than four months. On the other hand a shorter treatment period, as reported by Portuguese and Lipe, might be insufficient as one of their eculizumab-treated patients stayed on hemodialysis with this regime [22].

**Fig. 3:**
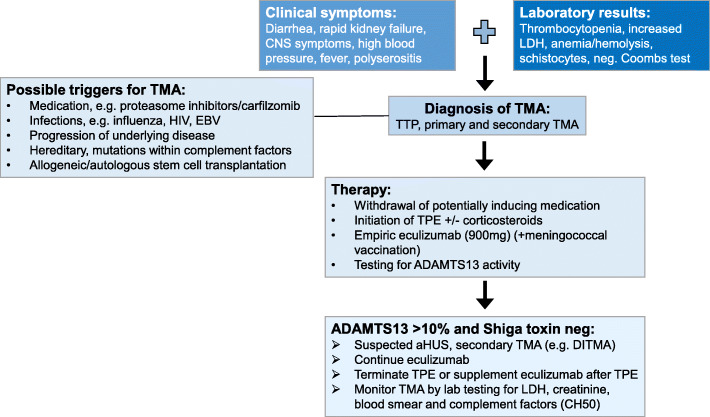
Guideline for diagnostic and therapeutic approach for TMA in MM: Whenever clinical and laboratory findings are consistent with TMA, one should rapidly terminate possible causative drugs, initiate therapeutic plasma exchange (TPE) and apply a first dose of eculizumab. As soon as ADAMTS13 activity is determined further therapeutic decisions can be drawn

TPE is generally recommended for TMA. Plasma exchange, however, is not without side effects, and can remove therapeutic antibodies from the circulation, thus its concurrent use is not recommended [6, 10]. Interestingly, in our cases the success of eculizumab was not fully dependent on the termination of TPE, since in patient #2 TPE was continued during the first two eculizumab doses. In this respect, concerns, that TPE would remove eculizumab from the blood or restore C5-levels available for activation, seem unfounded. On the other hand, a significant improvement of creatinine-levels and platelets was only achieved after termination of TPE in patient #2 (Fig. 1B). Thus, irrespective of the cause of TMA in MM patients under PI treatment or shortly after ASCT, eculizumab rather than TPE could be considered as standard first-line therapy [2, 6] (Fig. 3). Increased susceptibility to meningococcal infection during eculizumab treatment has to be considered and vaccination should be implemented [23]. As soon as ADAMTS13 activity is proven to be within the normal range (50–150%) and TTP is excluded, further treatment decisions can be drawn [6]. Additionally, in our experience short-term use of eculizumab was sufficient for effective TMA treatment. Furthermore, eculizumab treatment did not worsen MM parameters in neither of our patients.

Taken together, CFZ-induced TMA is a serious event and should be assumed in treated MM patients with rapid acute kidney failure and hemolysis. Fast treatment initiation with eculizumab can reverse pathological complement system activation and prevent organ damage. As shown in our patients, early initiated, short-term treatment with eculizumab can sufficiently terminate CFZ-TMA and counteract TMA-poor prognostic factors.

## Data Availability

Not applicable.

## Abbreviations

aHUS: Atypical hemolytic uremic syndrome
AKIN: Acute kidney injury network
ASCT: Autologous stem cell transplantation
BM: Bone marrow
CFZ-TMA: Carfilzomib-induced thrombotic microangiopathy
CR: Complete response
DITMA: Drug-induced thrombotic microangiopathy
FFP: Fresh frozen plasma
TMA: Thrombotic microangiopathy
TPE: Therapeutic plasma exchange
TTP: Thrombotic thrombocytopenic purpura
MM: Multiple myeloma
PC: Plasma cell
PI: Proteasome inhibitor
STEC: Shiga-toxin-producing E.coli
vgPR: Very good partial response
